# Supplementary figures and images for: Adiposity and body fat distribution based on skinfold thicknesses and body circumferences in Czech preschool children, secular changes
Source: PeerJ. 2024 Dec 13;12:e18695. doi: 10.7717/peerj.18695 (PMC11648688; doi:10.7717/peerj.18695)

Bland-Altman plot

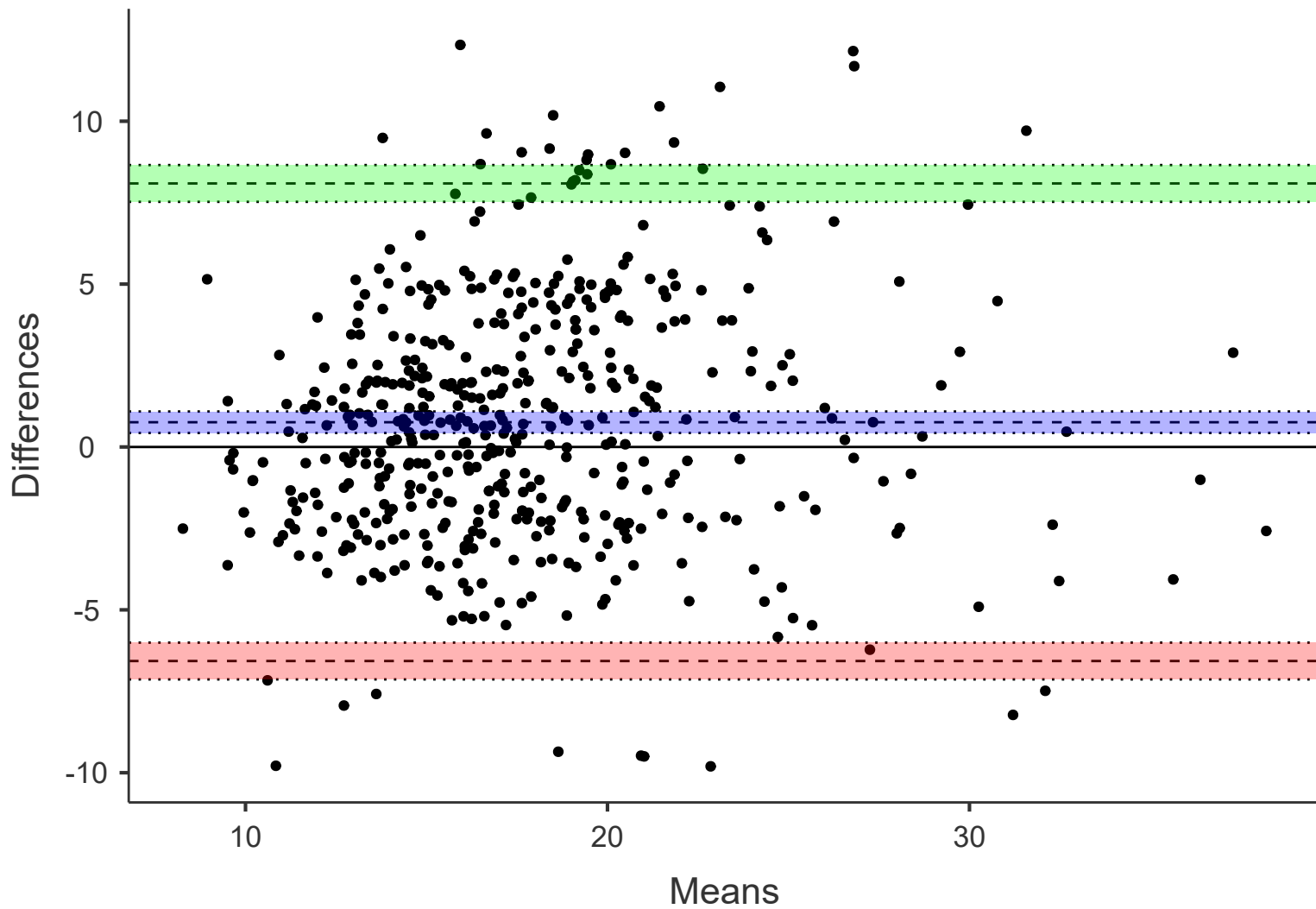

Supplement: Supplemental Information 1 — Bias evaluation for usability of body fat estimation in clinical practice with Blant– Altman method. [file peerj-12-18695-s001.pdf]

Bland-Altman plot

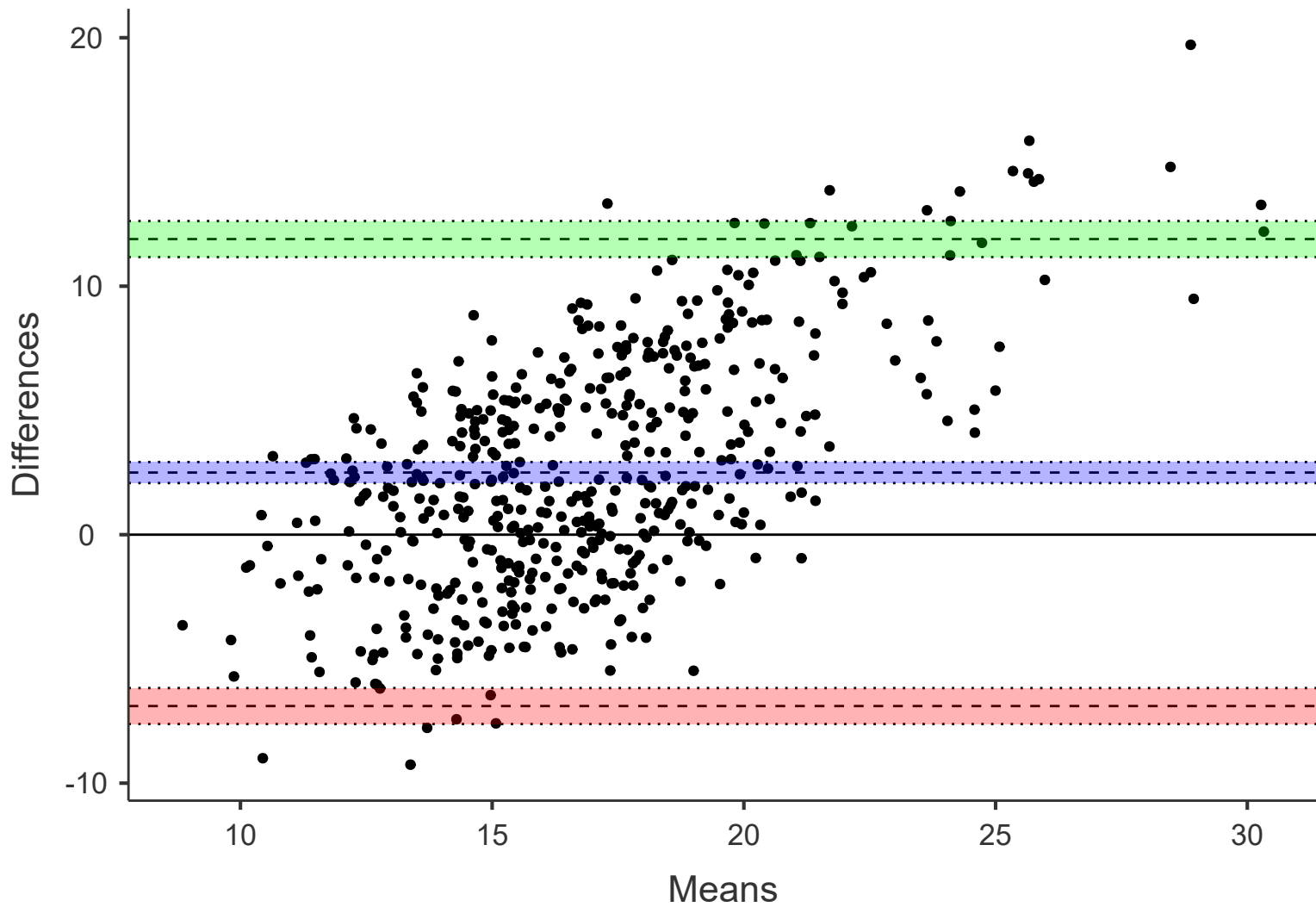

Supplement: Supplemental Information 2 — Bias evaluation for usability of body fat estimation in clinical practice with Blant– Altman method. [file peerj-12-18695-s002.pdf]

Bland-Altman plot

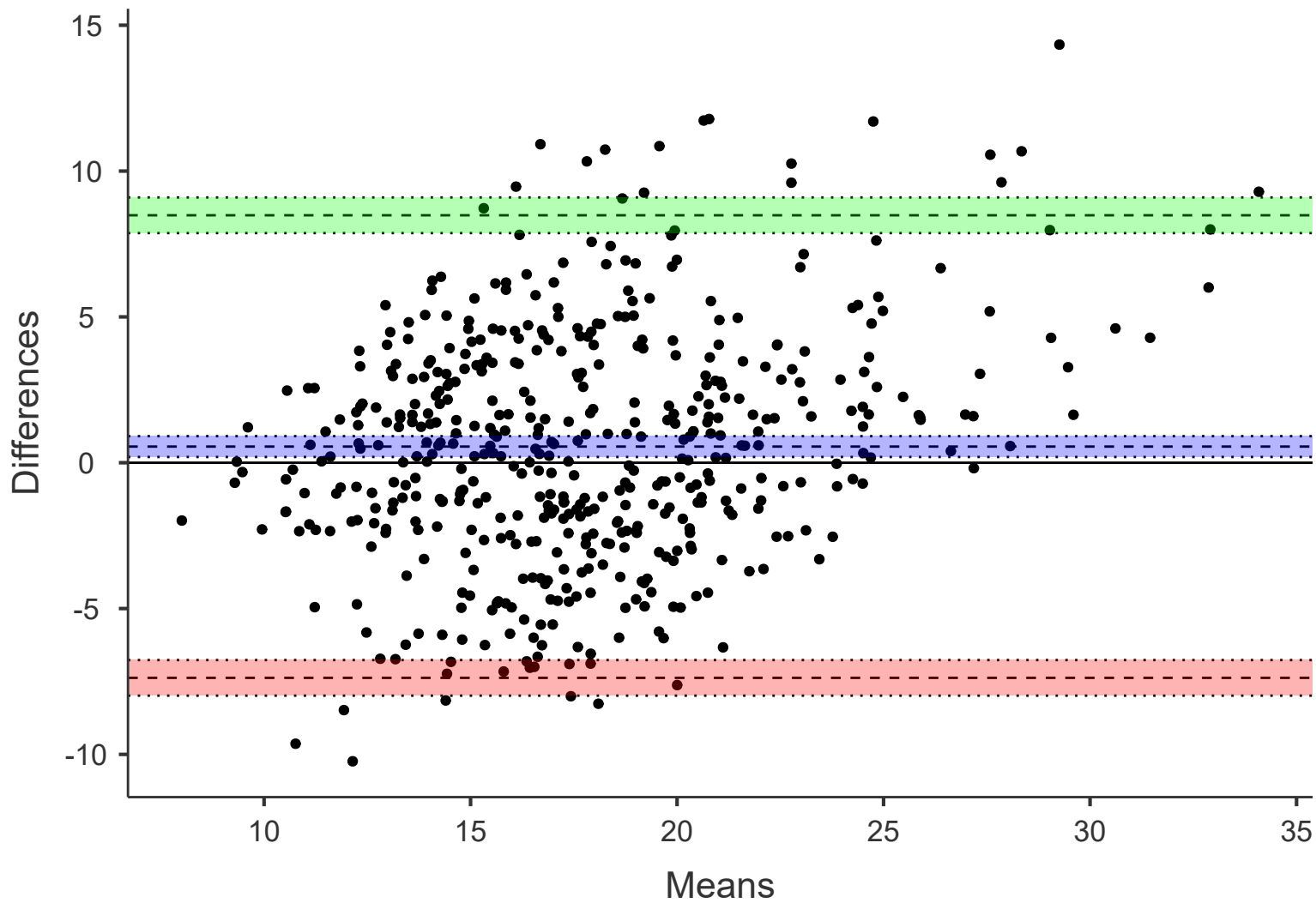

Supplement: Supplemental Information 3 — Bias evaluation for usability of body fat estimation in clinical practice with Blant–Altman method. [file peerj-12-18695-s003.pdf]
